# Urinary Incontinence in Female Athletes: A Systematic Review on Prevalence and Physical Therapy Approaches

**DOI:** 10.7759/cureus.64544

**Published:** 2024-07-14

**Authors:** Fizzia Syeda, Unnati Pandit

**Affiliations:** 1 Department of Community Health Physiotherapy, D. Y. Patil School of Physiotherapy, D. Y. Patil University, Navi Mumbai, IND

**Keywords:** nulliparous women, stress urinary incontinence, pelvic floor muscle training, physical therapy, female athletes, urinary incontinence

## Abstract

Urinary incontinence (UI) is an involuntary leakage of urine and is classified as stress, mixed, or urge. It is more common in females due to anatomical and physiological body differences. Moreover, the literature remarks an evident presence of UI with high-intensity physical activities. Therefore, the present integrative systematic review focused on the studies aimed at investigating the prevalence of UI in nulliparous sportswomen, studies illustrating sport-specific prevalence of UI, and studies demonstrating the impact of physical therapy intervention on UI. A literature search was carried out systematically on electronic databases consisting of Cochrane and Google Scholar databases from 2018 to December 2023. The keywords utilized to perform the literature search and include relevant articles consisted of “urinary incontinence,” AND “nulliparous,” AND “sportswomen,” OR “female athletes,” AND “physical therapy”. A total of nine studies were included in the present systematic review. The quality assessment of the studies was performed by using a measurement tool to assess systematic reviews (AMSTAR 2) scale, and the Mixed Methods Appraisal Tool was used for cross-sectional and randomized controlled trial studies. The data extracted included first author and year of publication, study design, sample or number of individuals involved in the study, age range of the participants, type of UI, type of sports involved, purpose of the study, methodological part, outcome measures derived, conclusion, and quality assessment of the studies. The review concluded that nulliparous athletes, especially those participating in high-impact activities, have a significant prevalence of UI. In addition, the physical therapy intervention consisting of pelvic floor muscle training (PFMT) along with education about pertinent pelvic anatomy was mostly performed on female athletes for the prevention and management of UI.

## Introduction and background

The International Continence Society (ICS) defines urinary incontinence (UI) as "the complaint of any involuntary leakage of urine." UI is categorized into three types: urge, stress, and mixed [1]. Leakage of urine can be comprehended by knowing its prevalence, severity, aggravating factors, impacts on social life and the quality of life, and the preventive measures taken by a person [2]. The prevalence of stress urinary incontinence (SUI) is the highest compared to urge and mixed incontinence, and research reveals that its incidence increases in the fifth decade of life [3]. Globally, UI is more common in females as compared to males due to their anatomical and physiological body differences. The prevalence of UI among females is 20%-50%, while in men it is only 3%-11% [4]. UI is not just a problem for older adult females but also a disturbing condition affecting young and middle-aged females. The onset of UI is most common in middle and late adulthood, with ages ranging from 30 to 79 years, but now it is getting more common in early adult females too. Many researchers have found an association between UI, with an increase in age and body mass index (BMI), childbirth and mode of deliveries, hysterectomy, heart disease, asthma, arthritis/rheumatoid arthritis, and level of physical activities and sports [5-9].

Though nearly all women and many healthcare professionals consider absolute UI as normal, however, evidence suggests that UI during stressful physical activity is common among young, physically active women even in the absence of known risk factors for incontinence [4,10]. Researchers suggest that this condition is not openly discussed by athletes, and they remain hesitant to be examined by experts [11]. Therefore, athletes with UI utilize their methodologies and try to adjust their problems according to their convenient approaches, such as preventative urination, avoidance of fluid intake, and wearing absorbent pads. However, they do not usually look for medicines, proficient exhortations, or doctor suggestions. As a result, many of the athletes quit their sports activities [12]. More research is needed to find out the prevalence and increase awareness of UI among athletes while participating in their sports activities and to design specific interventions. Therefore, the present integrative systematic review focused on the studies aimed at investigating the prevalence of UI in nulliparous sportswomen, studies illustrating sport-specific prevalence of UI, and studies demonstrating the impact of physical therapy intervention on UI.

## Review

This systematic review was conducted as per the Preferred Reporting Items for Systematic Reviews and Meta-Analyses (PRISMA) guidelines [13].

Data sources and search strategy

A systematic literature search was carried out on electronic databases consisting of Cochrane and Google Scholar databases from 2018 to December 2023. The keywords utilized to perform the literature search and include relevant articles consisted of “urinary incontinence,” AND “nulliparous,” AND “sportswomen,” OR “female athletes,” AND “physical therapy”.

Study screening and selection

For screening, the inclusion criteria consisted of studies involving women of age ≥18 years, female athletes or sportswomen experiencing UI along with physical therapy management, studies published between 2018 and 2023, studies published in the English language, and studies with full-text availability. However, studies that involved male participants or both sexes, study designs that consisted of case reports, commentaries, guidelines, editorials, book chapters, and letters to editors, studies not described in the English language, full-text non-availability, providing insufficient information related to the context were excluded.

The articles were evaluated by two reviewers independently to ascertain their suitability for inclusion in the review. First, for the removal of the duplicates, screening was performed based on the titles and abstracts. Secondly, the articles that were selected were screened again to remove articles not following the eligibility criteria. Finally, the selected articles were screened based on the full text to determine eligibility. Any discrepancies or disagreements among the reviewers were settled through consensus and discussions.

Data extraction

The data were extracted independently by the authors from the articles that included first author, year of publication, study design, sample or number of individuals involved, age range of the participants, type of UI, type of sports involved, purpose of the study, methodological part, outcome measures derived, and conclusion, and quality assessment of the studies highlighting the UI prevalence in female athletes along with the impact of the physical therapy management on UI. The authors combined and reviewed the data for inclusion in the systematic review.

Quality assessment

The methodological quality assessment of the included studies was performed by using a measurement tool to assess systematic reviews (AMSTAR 2) scale [14]. Moreover, the Mixed Methods Appraisal Tool (MMAT) was primarily used to appraise the methodological quality of qualitative research, randomized controlled trials (RCTs), non-randomized studies, quantitative descriptive studies (cross-sectional studies), and mixed methods studies [15,16]. The studies were graded as high, low, or of moderate quality based on the tools used for the respective studies.

Data synthesis

The critical narrative technique was employed to synthesize the results of the included studies. The narrative synthesis is described as the use of text, tables, and figures to summarize and validate study findings [15]. Higher methodological-quality studies incorporated study limitations, potential biases, and other factors into consideration during the analysis of the findings, offering a critical perspective for the review. As the number of relevant studies was limited, a meta-analysis approach or statistical synthesis was not suitable. Various research methodologies, physical therapy techniques, and outcome measures were employed in the included studies, leading to a significant degree of heterogeneity.

Results

**Figure 1:**
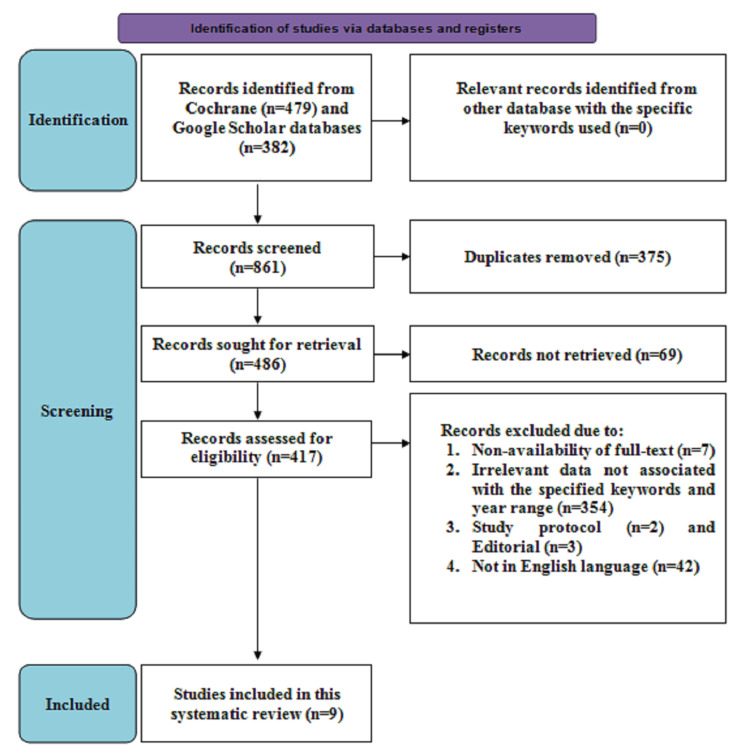
PRISMA flowchart illustrating search strategy. PRISMA: Preferred Reporting Items for Systematic Reviews and Meta-Analyses.

Figure 1 depicts the search strategy. Initially, 861 articles were screened, consisting of 479 studies from the Cochrane database and 382 articles from the Google Scholar database. The duplicate articles, consisting of 375 in number, were removed, after which 486 remained and were evaluated for retrieval, of which 69 were not retrieved. Following this, 417 articles were screened for eligibility, of which 354 provided irrelevant data associated with the specified keywords; seven articles reported the non-availability of the full text; two were study protocols; three were editorials; and 42 were not described in English and therefore were excluded. Hence, a total of nine studies consisting of three systematic reviews, one meta-analysis, one randomized controlled trial (RCT), and four cross-sectional studies describing UI prevalence in sportswomen or female athletes and the impact of physical therapy intervention on UI were included in this systematic review.

Moreover, the summary of the extracted data consisting of the first author and publication year, number of participants, age range of the participants, and type of UI and sports involved from the nine studies included is described in Table 1.

**Table 1:** Patient characteristics of the included studies. SUI: stress urinary incontinence, UUI: urge urinary incontinence, MUI: mixed urinary incontinence.

S. no	Author and year	Study design	Number of participants	Age range (years)	Type of urinary incontinence	Types of sports
1.	Almousa et al. (2019) [17]	Systematic review	18-503	18-45	SUI, UUI, MUI	Gymnastics, tennis, field hockey, softball, golf, basketball, swimming, track, volleyball, cheerleading, track and field, soccer, running, handball, water aerobics, weight lifting, cross-country skiing, artistic gymnastics, judo, trampoline, boot camp/CrossFit, rowing, cycling, pilates, and dance
2.	Dos Santos et al. (2018) [18]	Cross-sectional study	50	≥18	SUI, UUI, MUI	Low impact: dancing, rowing. High impact: basketball, athletics, futsal, gymnastics, running, cross fit, karate, volleyball, and taekwondo
3.	Khan (2019) [4]	Cross-sectional study	373	18-30 years	SUI, UUI, MUI	Athletics, volleyball, basketball, badminton, swimming, table tennis, tai-chi, cricket, indoor football, and throwball
4.	Mahoney et al. (2023) [19]	Cross-sectional study	425	>18	SUI	Powerlifting, weightlifting, strongman
5.	Pires et al. (2020) [20]	Systematic review and meta-analysis	1254	18-45	SUI, UUI, MUI	Volleyball, handball, basketball, athletics (track and field), indoor football, running, cross-country skiing
6.	Kelecic et al. (2023) [21]	Pilot cross-sectional observational study	70	18-36	SUI, UUI, MUI	Handball, athletics, weightlifting, gymnastics, alpine skiing, soccer, volleyball, swimming, table tennis, court tennis, water polo, and synchronized swimming
7.	Pires et al. (2020) [22]	Randomized controlled pilot study	13	≥18	SUI, UUI, MUI	Elite female volleyball athletes
8.	Sorrigueta-Hernández et al. (2020) [23]	Meta-analysis	The mean of controls was 85.49±175.23 per study and the mean of athletes per study was 284.38±373.86.	18-30	SUI, UUI, MUI	Low-impact sports included golf, throwing athletics, swimming, running athletics, and non-competitive sports. Moderate impact activities consisted of field hockey, skiing, cross-country, badminton, baseball, and tennis. High impact involved artistic gymnastics, gymnastics, soccer, ballet, jump sports, aerobics, judo, basketball, volleyball, handball, and rhythmic gymnastics.
9.	Garrington et al. (2022) [15]	Systematic review	302	18-40	SUI, UUI, MUI	Military, collegiate athletes, volleyball players

Furthermore, a detailed description regarding the purpose, methodology, results, and quality assessment of the nine studies included is demonstrated in Table 2.

**Table 2:** Detailed description of the included studies. UI: urinary incontinence, AI: anal incontinence, POP: pelvic organ prolapse, SUI: stress urinary incontinence, MUI: mixed urinary incontinence, UUI: urge urinary incontinence, PFMT: pelvic floor muscle training, G1: group 1, G2: group 2, G3: group 3, ICIQ-UI SF: International Consultation on Incontinence Questionnaire-Short Form, FSD: female sexual dysfunction, FSFI: The Female Sexual Function Index, FA: female athletes.

S no.	Author and year	Purpose	Methodology	Results	Quality assessment
1.	Almousa et al. (2019) [17]	To investigate the prevalence of UI in nulliparous female athletes by systematically reviewing the studies.	Databases consisting of Embase, Medline, Cochrane Library, and Cinahl were searched for literature review and the quality was assessed of the included studies. The data was extracted in a standardized data extraction spreadsheet.	The systematic review contained 23 studies. The range of the UI prevalence observed during sports activities was 5.7% to 80%. Depending on the sport, UI prevalence varies. UI was found to be most prevalent among trampoline athletes. The data indicate that female athletes, particularly those who participate in high-impact sports, frequently experience UI.	High
2.	Dos Santos et al. (2018) [18]	To determine the UI prevalence and FSD and analyze the risk factors in nulliparous athletes.	A cross-sectional study involving 50 nulliparous athletes was carried out. Athletes who competed at the municipal or state level and were at least eighteen years old, sexually active, in the reproductive phase and nulliparous were included. The team coaches conducted the athlete recruitment through phone or email. The athletes were given three questionnaires: demographic; ICIQ-UI-SF for UI; and FSFI, which assessed sexual function in six domains: sexual arousal, sexual desire, orgasm, vaginal lubrication, pain, and sexual satisfaction.	A 48% prevalence of UI was observed, of which 50% showed UUI, 37.5% showed SUI, and 12.5% showed MUI. FSD was present in 44% of cases. Twenty-four percent of athletes had both UI and FSD concurrently. Hours of training were revealed to be a risk factor for UI. Hence, the most susceptible to UI are nulliparous athletes who engage in high-impact modalities.	High
3.	Khan (2019) [4]	To investigate the frequency of urinary incontinence among female athletes	Non-probability purposive sampling technique was used. The duration of the study was from September 2017 to February 2018. Participants were enrolled in various sports academies. A self-administered questionnaire was the mode of investigation. The questionnaire included close-ended questions in three parts. Part A included demographics and menstrual history, suggestive past medical history; part B included questions related to sports activities, involving the time duration of physical activity, drug intake, and addictions; and part C included questions related to urinary incontinence that if the athlete was found to have urinary incontinence, would she consider it as a problem, what are the preventive measures she may have been taking, and whether she thinks that physical therapy would be a better option for treatment.	A total of 373 female athletes were included in the study. UI was experienced at least once by 242 (64.9%) athletes, while 131 (35.1%) had not experienced it. Out of this percentage, 12.1% had SUI, 36.7% had UUI, and 16.1% had MUI.	High
4.	Mahoney et al. (2023) [19]	To evaluate the frequency, acceptance, preferred information sources, and treatment rates in female athletes associated with SUI.	A novel cross-sectional survey was distributed online through social media groups among female strength athletes and responses were received from 425 women in less than four days.	50.2% reported experiencing incontinence during competition, 59.1% of athletes reported experiencing incontinence with regular strength training, and 43.5% reported experiencing incontinence with daily activities. Just 9.4% of the athletes who had incontinence had ever sought treatment, and 61.4% never had the condition prior to beginning their sports activity. According to 67.9% of all athletes surveyed, incontinence is a common occurrence in their sport. Hence, the findings indicate that female strength athletes frequently have SUI, which could be a result of the activity itself. Normalization of SUI is typical, and only a few athletes pursue treatment.	High
5.	Pires et al. (2020) [20]	To identify which modality is most likely to emphasize SUI and to organize the data that assessed the UI prevalence in female athletes.	Utilizing the EMBASE, PubMed, Web of Science, and Scopus databases, a comprehensive literature search of recent interventional trials of SUI over the previous ten years was conducted from September to December 2018. The Downs and Black scale were utilized to evaluate the methodological quality, and meta-analysis was employed to analyze the data gathered from various research.	Nine studies were included based on the eligibility criteria highlighting UI in various sports. The UI prevalence was 25.9% in female athletes in different sports, of which SUI involved 20.7%. The high-impact sport that was found to be the most prevalent was Volleyball (75.6%).	High
6.	Kelecic et al. (2023) [21]	To evaluate the frequency, risk factors, and severity in Croatian female athletes for UI.	Individual female athletes received an anonymous survey through online mode consisting of an ICIQ-UI SF questionnaire and general characteristics of sports in May 2022.	Among the 70 female athletes who competed in 12 different sports, the percentage of athletes with UI was 24.3%. This included 3 (42.9%) tennis players, 8 (29.6%) handball players, 2 (40%) water polo players, 1 (14.3%) synchronized swimmer, 7 (14.3%) soccer players, 1 (50%) weightlifter and the only (100%) swimmer. According to the ICIQ-UI SF, 70.59% of FA with UI reported having minor urine leaks once a week or less, mostly after clothing and urinating (35.29%). While the UI intensity ranges from minimal (47%) to moderate (53%), its interference with quality of life is mild (70.59%) and moderately severe (29.41%).	High
7.	Pires et al. (2020) [22]	To determine the impact of pelvic floor muscle training and its effectiveness in elite female volleyball athletes for SUI.	Fourteen athletes in the age range between 18 and 30, both continent and incontinent, were randomized to either the experimental or control groups. For four months, the experimental group followed a regimen to strengthen their pelvic floor muscles. There were three stages to this: power, strength training, and awareness/stabilization. During the same period, there was no intervention planned for the control group. For both groups, measurements were taken at the beginning and end of the study. A perineometer was used to measure maximum voluntary contractions, a Pad test was used to measure involuntary loss of urine, and the King's Health Questionnaire was used to assess quality of life.	The baseline anthropometric and sociodemographic features did not differ significantly. When the two groups were compared, the experimental group showed significant differences in the variation between the initial and final phases. It also reduced urine loss (p = 0.025) and improved maximum voluntary pelvic contractions (p < 0.001). The experimental group's percentage of urine loss dropped from 71.4 to 42.9%, indicating that athletes with SUI may benefit from the 16-week protocol intervention.	High
8.	Sorrigueta-Hernández et al. (2020) [23]	The objective of this study is to determine the scientific basis for pelvic floor dysfunctions that are linked to UI in female professional athletes and to assess if pelvic floor physiotherapy (PT) can effectively address UI in these athletes.	The study consisted of a meta-analysis in which the articles were analyzed using the keywords "pelvic floor dysfunction elite female athletes," "urinary incontinence elite female athletes," "pelvic floor dysfunction elite female athletes physiotherapy," and "urinary incontinence elite female athletes physiotherapy." The measures studied consisted of study design, sample size, age range, type of sport, prevalence of UI type diagnosed in athletes, etiopathogenesis, response to the PT and general treatment, and associated diseases or health conditions. Based on the impact of each sport on the pelvic floor the groups were divided into the following categories. G1: low-impact (swimming, throwing athletics, golf, running athletics, non-competitive sports); G2: moderate impact (field hockey, badminton, baseball, tennis, cross-country skiing); and G3: high impact (artistic gymnastics, aerobics, soccer, rhythmic gymnastics, ballet, gymnastics, judo, jump sports (high, long, triple and pole jump)), volleyball, basketball, handball).	The average number of athletes per study was 284.38±373.867, the mean age was 22.69±2.70. Case-control studies accounted for 39.60% of all study types, with cross-sectionals accounting for 30.20%. The study found that the most common types of UI were most often unspecified (47.20%), SUI (24.50%), or general UI (18.90%). 54.70% of studies were based on prevalence, followed by etiopathogenesis consisting of 28.30% and 17.00% on treatment.	Moderate
9.	Garrington et al. (2022) [15]	To assess the effectiveness and safety in elite female athletes and military personnel of specific prevention and management approaches for UI, AI, and POP.	Databases were searched using keywords like "female," "military," "athlete," and "pelvic floor dysfunction," related to the management and prevention of AI, UI, and POP. Two reviewers selected and evaluated the studies independently. After data extraction, a critical narrative synthesis method was performed.	A total of eight studies were included based on the criteria. One study was based on AI and seven on UI were conducted. Research on female athletes and military women who experienced UI symptoms frequently found that PFMT was a common and helpful treatment. Female athletes benefited from education. Concerningly, the self-management techniques involved the use of pads and fluid restriction.	High

Discussion

The UI prevalence ranges from 5.7% to 80%, as reported by the findings of the present systematic review, and this varies based on the type of sport. The highest prevalence was observed for trampolinists, volleyball players, and high-impact sports activity players. The UI is considered the most common PFM dysfunction in female athletes, affecting 15 to 17% of women every day [24,25]. Several studies also found a high prevalence of UI among female athletes [17,18]. There is a strong relationship between physical activity and SUI [26,27]. A possible justification for these higher rates is that intense physical activity promotes an increase in intra-abdominal pressure, and the repetitive increases may lead to weakness and stretching of the PFM and, consequently, to UI [18]. This increase in abdominal pressure results in morphologic and functional modifications, such as the deformation of ligaments and connective tissue [28,29]. This is believed to be the cause of urinary dysfunctions in young and nulliparous women who have no other risk factor when they reach the pressure threshold on the PFM [20,30]. However, several studies also showed that intense physical activity could strengthen the PFM through the co-contraction between them and the abdominal muscles [18,31].

In contrast, Da Roza et al. investigated the structural variations in the pelvic floor of nulliparous, without UI female athletes. At the mid-vaginal level, a thicker pubovisceral muscle was found in the incontinent group, depicting that SUI was related to the reduced response or delayed reaction and not to the strength or displacement of the pubovisceral muscle. The authors hypothesize that the muscle thickens in an attempt to counteract the sphincter urethrae's diminished reactivity because the pubovisceral muscles and sphincter urethrae are a part of the diaphragm. After years of athletic training, changes in the intrafusal fibers may be the cause of the delayed reaction [32].

Female athletes report a high prevalence of UI in high-impact sports [17,18,33]. Activities classified as high impact included many jumps and movements involving maximal contractions of the abdomen. These exert an impact force directly on the PFM and increase intra-abdominal pressure [18,25]. In the present systematic review, the highest prevalence was found for trampolinist players, volleyball players, sports involving jumping, artistic gymnastics, soccer, gymnastics, judo, ballet, rhythmic gymnastics, aerobics, handball, and basketball players. Similarly, some authors corroborate that the prevalence of UI ranges from 28 to 80%, with the highest prevalence in high-impact female athletes, such as trampolinists, gymnasts, hockey players, and ballet dancers [26,34,35]. The most common sport that can lead to UI is associated with jumping activity. Forty percent prevalence was found among all high-impact sports, which included team sports, track and field, and aerobics; Eliasson et al. found an 80% prevalence of UI among trampoline athletes [33], and Almeida et al. found that 37.5% of women who practiced jumps (an aerobic activity with repeated jumping) reported they had experienced UI [36].

Pelvic floor muscle training (PFMT) has been shown to alleviate UI symptoms and has been studied as a potential preventative and management strategy for UI. Army women and female athletes should execute these exercises at least three times a day for ten to fifteen repetitions. Women were also trained on the significance of PFMT and guided on how to include these exercises in home exercise programs. In addition to PFMT, female athletes reported using daily pads or involving fluid restriction as a self-management technique. These strategies may help in reducing or avoiding negative consequences associated with it such as frequent change of clothes and embarrassment [15,37]. 

Strengths and limitations

The results of this systematic analysis show that the risk of developing UI is higher in high-impact sports activities, and its prevalence may be higher in female athletes. Moreover, SUI may make it more difficult for women to engage in physical activity and sports, which would lower athletes' quality of life. Professionals will be able to execute preventative strategies and provide relevant advice on the prevention and intervention of UI if they have a thorough understanding of the impact of risk factor mechanisms on PFM. The review further demonstrated certain limitations, firstly, which involved no reporting of meta-analysis because of the heterogeneous nature of the methodological part of the studies included. The included studies' designs varied, and the data collection questionnaires used in each study were not the same. Furthermore, it is challenging to analyze the prevalence of UI for each sport in more detail because several studies only offered the prevalence rate of UI across a wide range of sports rather than independently for each sport. Secondly, it involved a small number of studies due to the selection criteria imposed. Additionally, research published in languages other than English was not included, which might have limited the number of relevant publications. Lastly, studies published in journals that are less indexed or published in databases other than those considered were excluded. Additionally, the results of this review highlight the need for more research using validated instruments that carefully account for response rate and selection bias to carefully evaluate the prevalence of UI related to sports-specific in nulliparous female athletes and to produce comparable results.

## Conclusions

The review demonstrated that nulliparous athletes, especially those participating in high-impact activity sports, have a significant prevalence of UI. Therefore, future research should determine how sustained high-impact exercise affects the pelvic floor and whether female development of UI can be prevented in female athletes. Additionally, it was observed that PFMT was the most common treatment performed for managing and preventing UI in female athletes when combined with education about important anatomy of the pelvis and PFMT technique and benefits. It is, therefore, essential for all healthcare professionals, fitness instructors, and coaches to have a thorough knowledge of the risk factor mechanisms impacting the PFM to implement preventative strategies or early diagnosis of UI. 
